# Rating of Perceived Exertion in Professional Volleyball: A Systematic Review

**DOI:** 10.5114/jhk/161614

**Published:** 2023-04-20

**Authors:** André Rebelo, João R. Pereira, Diogo V. Martinho, João Valente-dos-Santos

**Affiliations:** 1 CIDEFES, Centro de Investigação em Desporto, Educação Física e Exercício e Saúde, Universidade Lusófona, Lisboa, Portugal.; 2 COD, Center of Sports Optimization, Sporting Clube de Portugal, Lisbon, Portugal.; 3 Research Unity in Sport and Physical Activity (CIDAF, UID/DTP/04213/2020), Faculty of Sport Sciences and Physical Education, University of Coimbra, Coimbra, Portugal.; 4 Polytechnic of Coimbra, Coimbra Health School, Dietetics and Nutrition, Coimbra, Portugal.

**Keywords:** workload, team sports, athletes, monitoring

## Abstract

The rating of perceived exertion (RPE) is a non-invasive, cost effective, and time efficient strategy to measure training loads. However, data can be collected without following specific procedures and across a range of methods (e.g., different RPE scales and/or different operational questions). Consequently, practitioners working in professional volleyball can use this information in various ways with different assessment standards between them. Therefore, the purpose of the current review was to systematically and critically evaluate the use of RPE-based methods in professional volleyball athletes. Electronic searches were conducted in four databases (PubMed, SPORTDiscus, Scopus, and Web of Science). The electronic search yielded 442 articles, from which 14 articles were included in the systematic review. All included studies used the BORG-CR10 scale to calculate the session RPE. The main findings indicate that, to minimize the effect of the last exercise of the session, the athlete should be presented with the RPE question 10 to 30 minutes after the session is finished. Additionally, in order to evaluate the intensity of the training session, the question should be “how hard/intense was your session?”, avoiding questions without these adverbs or adjectives such as “how was your training session/workout?”. Future studies should analyse the collection of the localized RPE responses in professional volleyball athletes and their relationships with objective markers such as the number of jumps and accelerations.

## Introduction

Fatigue is a normal and desired part of the training process, and its severity can be observed as a continuum (Halson and Jeukendrup, 2004). When the proper balance between training stress and recovery is ensured, athletes experience acute fatigue in response to training sessions and recover within hours or days (Radojewski et al., 2018). However, if intense training continues without an adequate recovery period, athletes may enter a state of overreaching (Halson and Jeukendrup, 2004). When athletes experience a temporary reduction in performance levels as a result of training, they enter a state of functional overreaching (Meeusen et al., 2013). If training continues and unplanned fatigue persists, athletes may experience non-functional overreaching that can last for several weeks. The last phase of the fatigue continuum is called overtraining syndrome, which is characterized by decreases in performance levels that are usually accompanied by psychological disturbances that can remain for long periods (Meeusen et al., 2013). To prevent these maladaptations associated with excessive training loads, it is recommended that practitioners monitor training loads to ensure adequate recovery. In addition to these negative performance implications, excessive training loads increase the risk of injury and illness in high-performance athletes (Gabbett, 2010). These heightened risks demonstrate the importance of monitoring how athletes respond to training and competition, showing that the key for a good exercise prescription is an adequate understanding of the effect promoted by training loads on the human body (Busso, 2003).

Monitoring athletes’ training loads is better understood through sub-dividing loads into two types: internal and external (Halson, 2014). The internal training load (ITL) refers to the physiological stress that a training session induces in the athlete (Impellizzeri et al., 2005). The rating of perceived exertion (RPE) has become the most common method of monitoring the ITL as it is a non-invasive, cost effective, and time efficient strategy to measure training loads (Halson, 2014). The RPE method was originally developed by Borg (Borg, 1970), and Foster et al. (1995) created a simple technique to quantify the ITL using a modification of this scale. This technique is known as the session RPE (sRPE) and is derived by multiplying the overall RPE obtained at the end of a training session (or a match), using the Borg Category-Ratio 10 scale (BORG-CR10) by the total duration (in minutes) of the training session, to provide a modified training impulse (TRIMP) score.

Developing an understanding of the ITL response to specific mesocycles and the transition between mesocycles could inform future training prescription. However, RPE data can be collected without following specific procedures and across a range of methods (e.g., different RPE scales and/or different questions). For instance, in order to prevent that sRPE scores are overly influenced by how athletes felt at the end of the training session, the question should not be presented immediately after the session is finished (Foster et al., 1995). Consequently, practitioners working in professional volleyball can use this information in various ways with different scales and questions to assess this information. Therefore, a review of the literature specifically examining the available evidence and present suggestions to effectively monitor athletes with the RPE in professional volleyball would be of interest. Such a review can ensure that coaches would use quality information to prescribe training in applied settings. Thus, the purpose of the current review was to systematically and critically evaluate the use of RPE-based methods in professional volleyball.

## Methods

### Literature Search Strategy

Articles were systematically identified via four electronic databases (PubMed, SPORTDiscus, Scopus, and Web of Science) using the search strategy presented in Table S1 of the Supplementary File. The search string for each variable (the rating of perceived exertion and volleyball) was used independently, after which both were combined in the complete search strategy. The search was restricted to original peer-reviewed studies published in English, Spanish, or Portuguese with literature reviews and conference proceedings excluded. The search was developed to consider research articles published online or in print from the database inception until July 2022, when the search was conducted.

### Selection Criteria

The process for screening articles followed the Preferred Reporting Items for Systematic Reviews and Meta-Analyses (PRISMA) guidelines (Moher et al., 2015). The study protocol was registered in INPLASY (INPLASY202280034). Articles considered for inclusion in the review were those examining professional volleyball athletes and reporting RPE outcomes within, at least, one phase of the season (i.e., off-season, pre-season, or competitive period). The samples of participants consisted of volleyball athletes who were part of a professional team. Therefore, collegiate and young volleyball athletes were excluded from the present systematic review. Including experimental studies that implemented an intervention may have misrepresented the results, thus the review was restricted to cross-sectional or longitudinal observational study designs. Studies where player monitoring data were reported only during competitive games or during a portion of a phase of the season (e.g., one week) were excluded as they did not represent the complete workloads experienced by players during a specific period of the annual training plan.

Abstracts of all the articles identified in the search were screened independently against the pre-defined selection criteria by two authors (A.R. and D.V.M.). Any disagreements between the two authors regarding article inclusion were further discussed and, if agreement was not reached, a third author (J.R.P.) was consulted to establish consensus. Full-text copies were acquired for all papers that met title and abstract screening criteria. Full-text screening was performed by two reviewers (A.R. and D.V.M.). Again, any discrepancies were discussed until the authors reached an agreement and consulted a third author (J.R.P.) when required.

### Assessment of Methodological Quality

Methodological quality was assessed using a modified version of the Downs and Black (1998) checklist for assessing the methodological quality of healthcare interventions. This checklist had been validated for use with observational study designs (Downs and Black, 1998) and had been previously used to assess methodological quality in systematic reviews assessing cross-sectional and longitudinal studies (Fox et al., 2014, 2018). The number of items from the original checklist can be tailored to the scope and needs of the systematic review, with 10–15 items used in previous systematic reviews (Fox et al., 2014, 2018). For this review, 11 items in the checklist were deemed relevant (Table S2 of the Supplementary File). Each item was scored as “1” (yes) or “0” (no/unable to determine), and the scores for each of the 11 items were summed to provide the total quality score. The quality of each included article was rated against the checklist independently by two authors (A.R. and D.V.M.). Any disparity in the outcome of the quality appraisal was discussed, and a third author (J.R.P.) was consulted if a decision could not be reached.

### Data Extraction and Analysis

Data were extracted from each article by the lead author (A.R.). Data not provided or presented non-numerically were identified as “not reported”. The following data, where possible, were extracted from each article: (1) participants’ characteristics (sample size, sex, age, body height, and body mass); (2) monitoring period (i.e., seasonal phase(s) and duration); (3) objective measures (e.g., heart rate, time motion analysis); (4) RPE scale methods (e.g., scale, operational question).

## Results

### Search Findings and Study Selection

**Figure 1:**
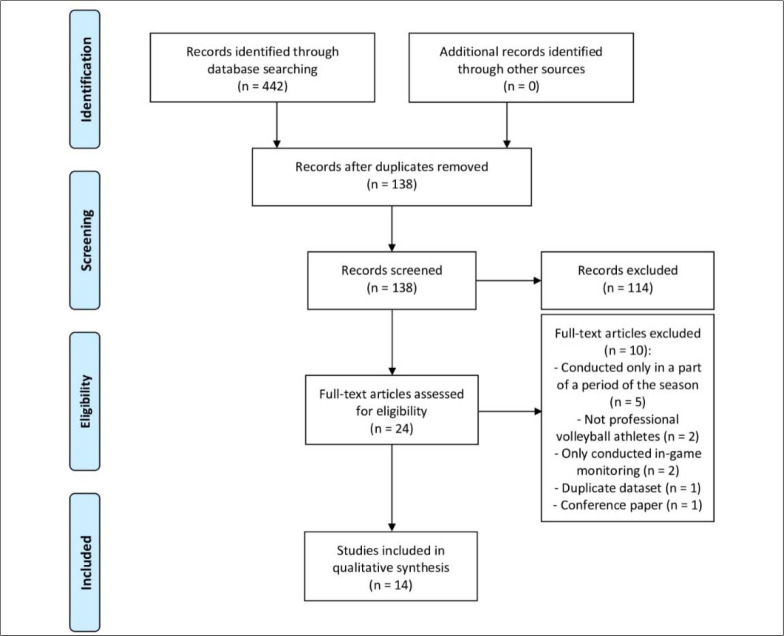
Preferred Reporting Items for Systematic Reviews and Meta-Analysis (PRISMA) flow diagram of the search strategy.

The electronic search yielded 442 articles (PubMed = 56, SPORTDiscus = 123, Scopus = 143, Web of Science = 120). A total of 304 duplicate records were removed, and further 114 irrelevant articles were excluded based on the title and the abstract; 24 full-text articles were screened and 10 were removed, leaving 14 articles for inclusion in the review. Reasons for exclusion were analysis only in a part of a period of the season (N = 5), non-professional athletes (N = 2), player monitoring limited to competitive games only (N = 2), duplicate data (N = 1), and a conference paper (N = 1). Full results of the search are presented in Figure 1.

### Methodological Quality

The ratings from the quality appraisal for each article are presented in Table S3 of the Supplementary File. Methodological quality scores ranged from 7 to 9 out of 11. In line with previous literature using the Downs and Black checklist (Fox et al., 2014, 2018), no articles were excluded based on methodological quality.

### Participant Characteristics

Characteristics of participants investigated in the included articles are presented in Table 1. Sample sizes ranged from 8 to 16 players. In total, 12 studies monitored only male and two monitored only female athletes.

**Table 1:** Participants’ characteristics of the included articles.

Study, year	N	Sex (M/F)	Age (years)	Body mass (kg)	Body height (cm)
Andrade et al., 2021	15	M	24 ± 4	96.67 ± 11.33	194.30 ± 6.65
Berriel et al., 2022	16	M	23.60 ± 4.93	92.10 ± 10.26	197 ± 6.29
Brandão et al., 2018	14	M	26.7 ± 5.5	95.8 ± 8.2	197 ± 7.9
Clemente et al., 2020	13	M	31.0 ± 5.0	88.9 ± 7.6	194 ± 7
Debien et al., 2018	15	M	24.0 ± 3.6	96.7 ± 11.3	194.3 ± 6.7
Domingos et al., 2022	11	M	26.4 ± 5.7	96.6 ± 9.0	197.6 ± 7.8
Duarte et al., 2019	14	M	24.0 ± 3.59	96.87 ± 9.85	194.36 ± 6.9
Horta et al., 2019	12	M	26.9 ± 4.6	94.9 ± 11.6	194.6 ± 8
Horta et al., 2020	9	M	26.4 ± 4.0	93.9 ± 5.7	198.9 ± 9.1
Lima et al., 2020	8	M	23.0 ± 5.22	84.5 ± 7.58	193.0 ± 9.71
Mendes et al., 2018	13	M	31 ± 5.0	88.9 ± 7.6	194 ± 7
Rabbani et al., 2021	13	F	25.8 ± 3.0	69.7 ± 7.6	178.1 ± 6.7
Timoteo et al., 2021	14	M	26.7 ± 5.5	95.8 ± 8.2	197.0 ± 7.9
Ungureanu et al., 2021	12	F	22 ± 4	74.1 ± 4.3	180 ± 6

### Collection of RPE Data

The duration of the selected studies was from six (Horta et al., 2019) to 36 weeks (Clemente et al., 2020; Debien et al., 2018; Mendes et al., 2018). Data were predominantly collected during preparatory and competitive periods (50%) (Andrade et al., 2021; Brandão et al., 2018; Debien et al., 2018; Duarte et al., 2019; Horta et al., 2020; Mendes et al., 2018; Timoteo et al., 2021). Three studies collected RPE-based data during the preparatory period only (21%) (Berriel et al., 2022; Domingos et al., 2022; Horta et al., 2019), and other three during the competitive period only (21%) (Clemente et al., 2020; Lima et al., 2020; Ungureanu et al., 2021). One study reported data during the transition period between clubs and national team camps (7%) (Rabbani et al., 2021). Only two studies complemented RPE-based ITL data with objective measurements such as inertial movement units (Lima et al., 2020) and the heart rate (Ungureanu et al., 2021). A detailed description of RPE data collection procedures is reported in Table 2.

**Table 2:** Data collection methods adopted to monitor the RPE in professional volleyball.

Study, year	Period	Duration	Objective measurements
Andrade et al., 2021	Pre + Comp	22 weeks	-
Berriel et al., 2022	Pre	10 weeks	-
Brandão et al., 2018	Pre + Comp	33 weeks	-
Clemente et al., 2020	Comp	36 weeks	-
Debien et al., 2018	Pre + Comp	36 weeks	-
Domingos et al., 2022	Pre	11 weeks	-
Duarte et al., 2019	Pre + Comp	35 weeks	-
Horta et al., 2019	Pre	6 weeks	-
Horta et al., 2020	Pre + Comp	19 weeks	-
Lima et al., 2020	Comp	15 weeks	IMU
Mendes et al., 2018	Pre + Comp	36 weeks	-
Rabbani et al., 2021	Transition	NR	-
Timoteo et al., 2021	Pre + Comp	27 weeks	-
Ungureanu et al., 2021	Comp	16 weeks	HR

Pre = preparatory period; Comp = competitive period; NR = not reported; IMU = inertial movement unit; HR = heart rate.

### Characteristics and Variables of the RPE Questionnaires

All included studies used the BORG-CR10 scale to calculate the sRPE (Table 3). “How was your training session?” was the most used question (29%) (Andrade et al., 2021; Brandão et al., 2018; Domingos et al., 2022; Duarte et al., 2019), followed by “how was your workout?” (21%) (Debien et al., 2018; Horta et al., 2019; Ungureanu et al., 2021). Four studies (29%) did not report the question that was used (Berriel et al., 2022; Mendes et al., 2018; Rabbani et al., 2021; Timoteo et al., 2021). Most studies included the weekly internal training load (wITL) in their results (79%) (Andrade et al., 2021; Berriel et al., 2022; Brandão et al., 2018; Clemente et al., 2020; Debien et al., 2018; Domingos et al., 2022; Duarte et al., 2019; Horta et al., 2019, 2020; Mendes et al., 2018; Timoteo et al., 2021), while few included derived variables such as training monotony (21%) (Clemente et al., 2020; Debien et al., 2018; Timoteo et al., 2021), strain (14%) (Debien et al., 2018; Timoteo et al., 2021), and the acute:chronic workload ratio (ACWR) (21%) (Clemente et al., 2020; Debien et al., 2018; Timoteo et al., 2021).

**Table 3:** Operational question and variables adopted in studies monitoring RPE-derived training loads in professional volleyball.

Study, year	RPE scale	Question	When was the question asked?	Daily-based variables	Weekly-based variables
Andrade et al., 2021	CR10	How was your TS?	30 min after	sRPE	wITL
Berriel et al., 2022	CR10	NR	15 min after	sRPE	wITL
Brandão et al., 2018	CR10	How was your TS?	30 min after	sRPE	wITL
Clemente et al., 2020	CR10	How hard was the TS?	30 min after	sRPE	wITL, monotony, ACWR
Debien et al., 2018	CR10	How was your workout?	30 min after	sRPE	wITL, monotony, strain, ACWR
Domingos et al., 2022	CR10	How was your TS?	30 min after	sRPE	wITL
Duarte et al., 2019	CR10	How was your TS?	30 min after	sRPE	wITL
Horta et al., 2019	CR10	How was your workout?	NR	sRPE	wITL
Horta et al., 2020	CR10	How did your training go?	30 min after	sRPE	wITL
Lima et al., 2020	CR10	How hard was the TS?	30 min after	sRPE	-
Mendes et al., 2018	CR10	NR	30 min after	sRPE	wITL
Rabbani et al., 2021	CR10	NR	30 min after	sRPE	-
Timoteo et al., 2021	CR10	NR	30 min after	sRPE	wITL, monotony, strain, ACWR
Ungureanu et al., 2021	CR10	How was your workout?	20 min after	sRPE	-

TS = training session; sRPE = session rating of perceived exertion; wITL = weekly internal training load; ACWR = acute:chronic workload ratio

## Discussion

In the present study, methods used to collect and interpret the RPE-based ITL in professional volleyball were reviewed. Articles considered for inclusion in the review were those examining professional volleyball athletes and reporting RPE outcomes within, at least, one phase of the season (i.e., off-season, pre-season, or competitive period), which may reflect the use of the RPE in practical settings. The findings of this systematic review provide the basis to establish a consensus regarding the practice adopted to collect and interpret the RPE in volleyball.

### Description and Quality of the Studies

The main deficiencies identified in the qualitative assessment concern the non-detailed description of estimates of the random variability in the data for the main outcomes. Moreover, all studies included had issues with their external validity. In order to reduce variations between subjects, the dataset should be organized as a whole (Bland and Altman, 1995) and, therefore, in this type of studies (i.e., longitudinal), ITL data should be reported according to the total number of training sessions/matches. Only four studies (Clemente et al., 2020; Debien et al., 2018; Mendes et al., 2018; Timoteo et al., 2021) (29%) included the match load in the ITL calculation, which represents an issue in the majority (i.e., 71%) of the studies, since the match day represents the most demanding day of the week (Clemente et al., 2020), and leaving it out of the analysis might skew the results. Besides that, the training prescription during the week can be supported by the matches’ ITL.

### Collection of RPE Data

Studies that reported results during the pre-season period showed that the loading pattern was defined by a progressive increase in the wITL between the first to the middle weeks of the pre-season, followed by a recovery week. Afterwards, there was another peak in the ITL, followed by a progressive decline towards the end of the preseason (Andrade et al., 2021; Berriel et al., 2022; Brandão et al., 2018; Domingos et al., 2022). However, in cases where the pre-season period was short, a progressive increase in the wITL was observed without any recovery week in the middle of this phase (Horta et al., 2019). This recovery week in the middle of the pre-season is often used to avoid unfavourable stress-recovery balance (Faude et al., 2011). However, it can be observed that in every scenario (i.e., short vs. long pre-season duration), a taper was implemented in the last weeks to better prepare for the start of the competitive phase to regain the training stimuli. Also, during this period, aerobic fitness is negatively correlated with the load perceived during the weeks following the test in professional volleyball players (Berriel et al., 2022). This finding indicates that the assessment and development of cardiorespiratory fitness is important to allow volleyball players to better tolerate training loads and avoid excessive fatigue (Freitas et al., 2014).

Studies conducted during the competitive period reported a wave distribution of the wITL (Andrade et al., 2021; Debien et al., 2018; Duarte et al., 2019). Due to various travels made and games played against teams of different levels, the number of training sessions reduced during the competitive period (Miloski et al., 2016). Therefore, this wave distribution of the training load can avoid a possible decrement in performance. This can be done by increasing training loads in weeks in which the team has a low possibility of winning or losing the game (Issurin, 2010; Miloski et al., 2016).

None of the studies included RPE collection in complementary training sessions (e.g., gym sessions). While, in the short term, this might not be that important, missing out this information in the accumulated ITL may bias some data, especially those related to spike variables, such as ACWR.

### Characteristics and Variables of the RPE Questionnaires

According to this systematic review, the BORG-CR10 is the “gold standard” approach to collect RPE data in professional volleyball as every study included this scale within their methods. However, some inconsistencies were observed in respect to RPE questions adopted, indicating the lack of a standard questionnaire. Only two studies reported the question “how hard was your training session?” (Clemente et al., 2020; Lima et al., 2020). This type of questions that include words such as “hard” or “intense” is better targeted at what is intended for the RPE (Rago et al., 2020) compared to most questions implemented in the included studies such as “how was your training session/workout?” or “how did your training go?” (Andrade et al., 2021; Brandão et al., 2018; Debien et al., 2018; Domingos et al., 2022; Duarte et al., 2019; Horta et al., 2019, 2020; Ungureanu et al., 2021). Also, some problems exist in the fact that four studies did not report the question used to collect RPE data (Berriel et al., 2022; Mendes et al., 2018; Rabbani et al., 2021; Timoteo et al., 2021).

The use of sRPE is more preferred within the professional volleyball environment than the use of derived variables such as monotony, strain, and ACWR. Previous studies have already shown that the TRIMP method fails in reflecting the demands of intermittent sports, the same way the mean of the heart rate in exercises of prolonged nature is impracticable and may not provide significant data, reinforcing the importance to use other methods to monitor the ITL in sports like volleyball, such as the sRPE (Robson-Ansley et al., 2009). Nevertheless, since the relationship between the training session duration and the perceived exertion in volleyball has not been investigated yet, practitioners should be cautious when implementing the sRPE method after long training periods as athletes can adopt a pacing strategy to be fresh by the end of the session. On the other hand, RPE data in short time sessions may be representative of the actual exercise intensity as opposed to objective measurements (e.g., distance covered or the number of jumps) (Eston, 2012) and should be used in volleyball high intensity practices.

It should also be noted that most studies (i.e., 79%) presented the RPE question to volleyball athletes 30 minutes after the training session was finished. This recommendation comes from the original concept of sRPE to prevent athletes’ responses to be highly influenced by the last portion of the training practice (Foster et al., 1995). However, this time delay required is often a limitation when working with high performance athletes as they are not always predisposed to wait that long to answer this question daily. Previous studies outside volleyball have demonstrated that the sRPE is temporally robust, showing that there are almost no differences between answering the question 10 or 30 minutes after the training session finishes (Uchida et al., 2014). Although future research still needs to assess the effect of measurement timing on s-RPE in professional volleyball athletes, these previous results further support the practical usefulness of the sRPE to measure the ITL in athletes.

### Limitations and Recommendations for Future Research

Although the present study focused on RPE collection practices, other factors such as the players’ position, competitive schedule, and types of exercises performed might have an impact on perceptual responses of professional volleyball athletes. For instance, results from previous studies have already shown that starter players presented a greater ITL in comparison to non-starters in all periods of the season (Horta et al., 2017). Also, during weeks with more than one game (i.e., congested weeks), volleyball players have a higher ITL compared to normal weeks with one game only (Brandão et al., 2018; Mendes et al., 2018). These training and match load subjective perceptions increase as the volleyball season progresses, highlighting the importance of training periodization (Pires and Ugrinowitsch, 2021). Moreover, it is well reported that volleyball players from different positions have different external training load (ETL) responses (e.g., the number of jumps, jumps’ height) (García-de-Alcaraz et al., 2020; Skazalski et al., 2018) and different perceptions of the ITL across the competitive phase (Ungureanu et al., 2021). Therefore, in professional volleyball, the players’ RPE is dictated not only by match/schedule related factors, but also by the ETL, and future reviews should take this into consideration.

Since the RPE represents a subjective perception of the effort from all organs, as well as the perception of fatigue and pain, it is sometimes insufficient to capture the whole range of exercise-related perceptual sensations, due to their generalization and oversimplification (Hutchinson and Tenenbaum, 2006). For this reason, previous studies have already proposed the differentiation between the muscular RPE and respiratory RPE (Ekblom and Golobarg, 1971; Pandolf, 1982). However, no study in professional volleyball has evaluated this localized RPE responses in these athletes, and future studies should consider this issue. Also, only two studies included objective markers in their methods alongside the RPE collection (Lima et al., 2020; Ungureanu et al., 2021). Previous research showed that the sRPE method was correlated with several objective markers of training loads. For instance, velocity at the lactate threshold and velocity at the onset of the blood lactate threshold were strongly correlated with the sRPE method during soccer training sessions (Akubat et al., 2012). Additionally, the number of impacts and the distance covered were also correlated with the sRPE method in rugby (Lovell et al., 2013).

Research in volleyball shows promising results when using inertial movement unit technology (e.g., VERT) to monitor jumping metrics, such as jump height and the jump count (Herring and Fukuda, 2022; Lima et al., 2020; Silva et al., 2019; Skazalski et al., 2018). This method of monitoring the ETL can be extremely useful, since there are significant differences in competition and in the training jump count, jump height and jump load between positions in female (Herring and Fukuda, 2022) and male volleyball athletes (García-de-Alcaraz et al., 2020; Kupperman et al., 2021; Skazalski et al., 2018). For example, outside hitters had the highest jump height followed by middle blockers and right-side hitters (Herring and Fukuda, 2022). Female (Herring and Fukuda, 2022) and male (García-de-Alcaraz et al., 2020; Skazalski et al., 2018) volleyball middle blockers showed a higher jump count and jump rate compared to outside hitters and right-side hitters. Therefore, future studies in professional volleyball should examine the correlations between the sRPE method and different objective markers, such as the jump count, jump height, the number of accelerations, and the number of high-speed runs. Finally, to the authors’ knowledge, there has been no study that collected the RPE during the off-season period. This could prove to be of extreme importance, especially for the calculation of the ACWR during the first weeks of the pre-season, which is often neglected since there are no previous data available for its calculation.

Among observational studies in professional volleyball, the use of RPE-based methods presents some inconsistencies. While most studies present the RPE question 30 minutes after the session is finished, the sRPE method appears to be temporally robust and, therefore, coaches can apply this method sooner if they wish to do so. Additionally, in order, to evaluate the intensity of the training session, the question should be “how hard/intense was your session?”, avoiding questions without these adverbs or adjectives such as “how was your training session/workout?” or “how did your training go?”. Future studies should analyse the collection of the localized RPE responses in professional volleyball athletes and their relationships with objective markers such as the number of jumps and accelerations. Finally, the off-season is a period that is being neglected in professional volleyball research, and future studies should analyse this phase of the season so that coaches can have data from the start of the pre-season from their athletes.

## Practical Implications

This review offers a few practical implications for sports practitioners. Athletes should answer the question “how hard/intense was your session?” 10 to 30 minutes after finishing the training session, using the BORG-CR10 scale, to better target at what is intended for the RPE. Professionals should aim to evaluate the localized RPE responses of volleyball athletes, as the RPE alone is insufficient to capture the whole range of exercise-related perceptual sensations. Finally, due to the intermittent nature of professional volleyball, the sRPE method should be employed to monitor the ITL over methods such as the TRIMP.

## Figures and Tables

**Table S1:** Search strategy used to locate relevant research articles.

Variable	Search terms
Rating of perceived exertion	(‘internal training load’ OR ‘workload’ OR ‘training impulse’ OR ‘training response’ OR ‘TRIMP’ OR ‘internal load’ OR ‘exposure’ OR ‘RPE’ OR ‘rating of perceived exertion’ OR ‘summated-heart-rate-zone’ OR ‘SHRZ’ OR ‘PlayerLoad’ OR ‘BodyLoad’)
Volleyball	‘volleyball athlete’ OR ‘volleyball player’
Training load AND volleyball (final search)	‘1 AND 2’

**Table S2:** Questions from the modified Downs and Black checklist used to evaluate methodological quality of the included articles.

Question no.	Question
	**Reporting**
1	Is the hypothesis/aim/objective of the study clearly described?
2	Are the main outcomes to be measured clearly described in the introduction or methods section?
3	Are the characteristics of the subjects included in the study clearly described?
4	Are the main findings of the study clearly described?
5	Does the study provide estimates of the random variability in the data for the main outcomes?
6	Have actual probability values been reported (e.g., 0.035 rather than < 0.05) for the main outcomes except where the probability value is < 0.001?
	**External validity**
7	Were the subjects asked to participate in the study representative of the entire population from which they were recruited?
8	Were those subjects who were prepared to participate representative of the entire population from which they were recruited?
	**Internal validity bias**
9	If any of the results of the study was based on “data dredging,” was this made clear?
10	Were the statistical tests used to assess the main outcomes appropriate?
11	Were the main outcome measures accurate (valid and reliable)?

**Table S3:** Results of methodological quality assessment for included articles.

Study	Downs and Black checklist question number											Total
	Reporting						External validity		Internal validity bias			
	1	2	3	4	5	6	7	8	9	10	11	
Andrade et al., 2021	1	1	1	1	0	1	0	0	1	1	1	**8**
Berriel et al., 2022	1	1	1	1	0	1	0	0	1	1	1	**8**
Brandão et al., 2018	1	1	1	0	0	1	0	0	1	1	1	**7**
Clemente et al., 2020	1	1	1	1	0	1	0	0	1	1	1	**8**
Debien et al., 2018	1	1	1	1	1	1	0	0	1	1	1	**9**
Domingos et al., 2022	1	1	1	1	0	0	0	0	1	1	1	**7**
Duarte et al., 2019	1	1	1	1	0	1	0	0	1	1	1	**8**
Horta et al., 2019	1	1	1	1	0	1	0	0	1	1	1	**8**
Horta et al., 2020	1	1	1	1	0	1	0	0	1	1	1	**8**
Lima et al., 2020	1	1	1	1	0	1	0	0	1	1	1	**8**
Mendes et al., 2018	1	1	1	1	0	1	0	0	1	1	1	**8**
Rabbani et al., 2021	1	1	1	1	0	1	0	0	1	1	1	**8**
Timoteo et al., 2021	1	1	1	1	0	1	0	0	1	1	1	**8**
Ungureanu et al., 2021	1	1	1	1	0	1	0	0	1	1	1	**8**

1 = yes; 0 = no/unable to determine
